# Inducible Costimulator Expressing T Cells Promote Parasitic Growth During Blood Stage *Plasmodium berghei* ANKA Infection

**DOI:** 10.3389/fimmu.2018.01041

**Published:** 2018-05-28

**Authors:** Gajendra M. Jogdand, Soumya Sengupta, Gargee Bhattacharya, Santosh Kumar Singh, Prakash Kumar Barik, Satish Devadas

**Affiliations:** Infectious Disease Biology, Institute of Life Sciences, Bhubaneswar, India

**Keywords:** malaria, inducible costimulator, T cells, IFN-γ, T-bet, CD4, CD8

## Abstract

The lethality of blood stage *Plasmodium berghei ANKA* (PbA) infection is associated with the expression of T-bet and production of cytokine IFN-γ. Expression of inducible costimulator (ICOS) and its downstream signaling has been shown to play a critical role in the T-bet expression and IFN-γ production. Although earlier studies have examined the role of ICOS in the control of acute blood-stage infection of *Plasmodium chabaudi chabaudi* AS (a non-lethal model of malaria infection), its significance in the lethal blood-stage of PbA infection remains unclear. Thus, to address the seminal role of ICOS in lethal blood-stage of PbA infection, we treated PbA-infected mice with anti-ICOS antibody and observed that these mice survived longer than their infected counterparts with significantly lower parasitemia. Anti-ICOS treatment notably depleted ICOS expressing CD4^+^ and CD8^+^ T cells with a concurrent reduction in plasma IFN-γ, which strongly indicated that ICOS expressing T cells are major IFN-γ producers. Interestingly, we observed that while ICOS expressing CD4^+^ and CD8^+^ T cells produced IFN-γ, ICOS^−^CD8^+^ T cells were also found to be producers of IFN-γ. However, we report that ICOS^+^CD8^+^ T cells were higher producers of IFN-γ than ICOS^−^CD8^+^ T cells. Moreover, correlation of ICOS expression with IFN-γ production in ICOS^+^IFN-γ^+^ T cell population (CD4^+^ and CD8^+^ T cells) suggested that ICOS and IFN-γ could positively regulate each other. Further, master transcription factor T-bet importantly involved in regulating IFN-γ production was also found to be expressed by ICOS expressing CD4^+^ and CD8^+^ T cells during PbA infection. As noted above with IFN-γ and ICOS, a positive correlation of expression of ICOS with the transcription factor T-bet suggested that both of them could regulate each other. Taken together, our results depicted the importance of ICOS expressing CD4^+^ and CD8^+^ T cells in malaria parasite growth and lethality through IFN-γ production and T-bet expression.

## Introduction

Malaria is a major cause of mortality in millions of infected individuals every year, especially children from developing countries. Among all human Plasmodium strains, infection with *Plasmodium falciparum* is the leading cause of death involving severity, cerebral manifestation, and multi-organ dysfunction. To some extent, murine infection of *Plasmodium berghei ANKA* (PbA) can be correlated to human *P. falciparum* infection corresponding to parasite growth, lethality, or severity and immune response (1). As human malaria studies are restricted to only clinical observations, modulation of T cell immune response in murine models can provide a better insight to ameliorate malaria pathology and vaccine design (2, 3). The role of T cells in malaria infection, however, has been controversial as studies have shown both its critical role in protection from the malaria parasite and its direct role exacerbating malaria pathogenesis. As an example, CD4^+^ T cells play a major role in the clearance of parasite *Plasmodium chabaudi chabaudi* blood stage infection (4). However, during lethal PbA infection, both CD4^+^ and CD8^+^ T cells are involved in cerebral manifestation. Moreover, depletion of both the T cells by antibodies before or during infection ameliorated pathology (5). Thus, these studies among others suggested that T cells play both protective as well as pathological roles during malaria infection.

In both human and murine malaria, CD4^+^ and CD8^+^ T cells are producers of IFN-γ, which have been shown to play crucial protective and pathological roles (6, 7). During lethal malaria infection, extraneous administration of IFN-γ led to the dose-dependent protection of BALB/c mice (8). In contrast, another study suggested that IFN-γ produced by CD4^+^ T cells enhanced CD8^+^ T cell accumulation in the brain leading to augmented cerebral malaria (9). Moreover, suppressing IFN-γ production from T-bet positive CD4^+^ T cells efficiently hampered parasite clearance (10). Also, studies with T-bet knockout mice have demonstrated the role of T-bet in regulating parasite burden as well as its role in pathogenesis during experimental cerebral malaria (11). Taken together, these studies indicated that both IFN-γ and T-bet play a role in malaria parasite growth and lethality.

Along with antigenic stimulation, signaling through CD28 plays a critical role in T cell-mediated immunity in clearance of acute blood-stage *P. chabaudi* infection (12). Inhibiting CD28 signaling by blocking CD86 with anti-CD86 antibody preferentially differentiated T cells toward IFN-γ producing Th1 cells and inhibited the Th2 cytokine IL-4. This Th1 cytokine IFN-γ controlled acute parasite infection but did not play a role in limiting chronic malaria infection. Thus, blockade of CD28 signaling suggested that IL-4 production during malaria infection required CD28 signaling whereas augmentation of IFN-γ illustrated the involvement of other co-stimulatory molecules (13). Moreover, *P. chabaudi* infection in the CD28 knock out mice also demonstrated that redundant CD28 signaling pathway with other costimulatory molecules might play a role in IFN-γ production (14). Thus, these studies suggested that IFN-γ production during malarial infection could involve other co-stimulatory molecules.

Inducible costimulator (ICOS), a CD28 homolog, plays a critical role in T cell proliferation, differentiation, cytokine secretion, cell–cell interaction, and B cell maturation (15–21). Depending on the nature of antigen and chronicity of infection, ICOS signaling mediates differential effector CD4^+^ T cell response. As an example, during *Schistosome mansoni* infection, Th2 and Tfh response were observed whereas, during *Toxoplasma gondii* and *Mycobacterium tuberculosis* infection (Mtb), it was a Th1 response (22–24). In contrast, during non-lethal blood stage *P. chabaudi chabaudi* infection, in the absence of ICOS, enhanced Th1 response led to reduced peak parasitemia (20). In case of CD8^+^ T cells, ICOS signaling played a role in its activation, expansion, and enhanced secondary response (21, 25). A substantial defect in antigen-specific CD8^+^ T cells along with hampered CD4^+^ T cells response was observed during Salmonella infection in ICOS knockout mice (26). Moreover, during late stage of Mtb, ICOS deficiency was associated with reduced Mtb-specific CD8^+^ T cell response (27). Taken together, these studies strongly suggest that ICOS plays a critical role in CD4^+^ and CD8^+^ T cell response in both intra and extracellular infection. However, during lethal intracellular blood-stage of PbA infection, the role of ICOS in T cell response (CD4^+^ and CD8^+^) remains unclear.

In this study, we examined the role of ICOS in parasite growth and lethality during lethal PbA infection in BALB/c mice. We characterized ICOS expression and found that both CD4^+^ and CD8^+^ T cells express higher ICOS in the PbA-infected mice. Upon depletion of ICOS-expressing T cells by anti-ICOS antibody treatment, we observed prolonged survival of mice with lower parasitemia, suggesting a positive role for ICOS expressing T cells in PbA parasite growth. Further, we report that these T cells are major producers of IFN-γ as correlated with reduced plasma IFN-γ cytokine level and depleted percentage of ICOS expressing T cells upon anti-ICOS treatment. We found both CD4^+^ and CD8^+^ T cells expressed transcription factor, T-bet, which is already known to be involved in malaria pathogenesis. Collectively, our study demonstrates that ICOS expressing CD4^+^ and CD8^+^ T cells are involved in malarial parasite growth and lethality through IFN-γ production and T-bet expression.

## Materials and Methods

### Ethics Statement

The use of animals and animal procedures were approved by the Institutional Animal Ethics Committee, Institute of Life Sciences, Bhubaneswar, India in accordance with the “Committee for the Purpose of Control and Supervision of Experiments on Animals (CPCSEA).”

### Mice, Antibodies, Kits, Reagents, and Malaria Parasite

Male BALB/c mice (6–8 weeks old) used for this study were maintained under pathogen-free condition at institutional animal house facility. Anti-mouse antibodies Alexa Fluor 700-CD8 (Clone 53-6.7), APC-cy7-CD19 (Clone 1D3), APC-cy7-CD45R/B220 (Clone RA3-6B2), were procured from BD Biosciences (San Jose, CA, USA), FITC-CD4 (clone GK 1.5), FITC CD278 (7E.17G9), APC-CD278 (Clone-C398.4A), PerCP-Cy 5.5-CD4 (clone RM4-5), PE-Cy7-IFN-γ (clone XMG 1.2), PE-T-bet (clone eBio 4B10), Alexa Fluor 700-CD8 (53-6.7) were from eBioscience (San Diego, CA, USA), Brilliant violet 605 TCR Vb (clone H57-597), APC-CD278 (clone C398.4A), Anti-CD49b (clone DX5) were from BioLegend (San Diego, CA, USA). Purified antibodies Anti-mouse CD278 ICOS (clone 7E.17G9), Anti-Rat IgG2b isotype control, and anti-CD16/32 were procured from BioXcell (West Lebanon, USA). Dynabeads untouched mouse CD4^+^ T cell isolation kit was procured from Life Technologies AS (Oslo, Norway) and CD8^+^ T cell negative selection kit was from Stem cell technologies (Vancouver, BC, Canada). Phorbol 12-myristate 13-acetate (PMA), Ionomycin, Brefeldin A was procured from Sigma-Aldrich (St. Louis, MO, USA). Malarial parasite *P. berghei ANKA* (MRA-671, MR4, ATCC, Manassas, VA, USA) was obtained from MR4 repository, ATCC, Manassas, VA, USA.

### Parasite Infection

*Plasmodium berghei* ANKA parasitized red blood cells were stored in liquid nitrogen. The parasitic infection was initiated by thawing *P. berghei* ANKA parasite stabilate and intraperitoneally (i.p.) injecting into a donor mouse. After initial expansion of parasites in the donor mouse, blood was collected from tail vein bleed and serially diluted in PBS. Infection was then induced in experimental mice by intravenous (i.v.) injection of 1 × 10^4^ *P. berghei* ANKA parasitic RBCs. After 3 days of infection, percent parasitemia was enumerated by thin blood films stained with modified Giemsa stain (Sigma-Aldrich, St. Louis, MO, USA).

### *In Vivo* Depletion of ICOS-Positive T Cells

On day 1 of PbA infection, ICOS positive T cells were depleted by administering a single dose of anti-CD278 monoclonal antibody (clone 7E.17G9) and its isotype control (rat IgG2b) at 0.2 mg dose, *via* intraperitoneal (i.p.) injection in 200 µl DPBS. Specific depletion of ICOS positive T cells was analyzed by flow cytometry as shown in figure (Figure 3H).

### Determination of Plasma Cytokines

On day 3, day 5, and day 7, day of PbA infection, murine blood was collected in 15% acid citrate dextrose anticoagulant and centrifuged at 1,000 *g* for 10 min. The collected plasma was stored in −80°C until assayed for cytokines. Cytokine from stored plasma was quantified using the Milliplex mag mouse cytokine/chemokine kit as per the manufacturer’s protocol (Millipore, Billerica, MA, USA). The samples were acquired in Bio-plex200 system, and the concentration of cytokines was calculated using Bio-Plex manager software with a five-parameter curve-fitting algorithm applied for standard curve calculation.

### Intracellular Cytokine Staining

For measuring T cell secreted cytokines, the negatively selected splenic CD4^+^ T cells (purity was >93%) and splenocyte (for CD8^+^ T cell analysis) from uninfected and PbA-infected mice were cultured in RPMI 1640 complete medium [RPMI 1640 (# P04-16500), PAN-Biotech GmbH, Germany] supplemented with 10% FBS US origin (#1302-P100402, PAN-Biotech GmbH, Germany), 50 µM 2-ME, 100 U/ml penicillin and 100 µg/ml streptomycin (# P4333-100 ml, Sigma-Aldrich, USA) and stimulated by 20 ng/ml PMA/1 μg ionomycin for 5 h after with Brefeldin A added in the last 2.5 h of stimulation. Stimulated cells were stained with dead cell discrimination dye for 20 min on the ice and then washed and stained for surface markers. Surface stained cells were fixed with Fixation/permeabilization buffer of BD Biosciences for 20 min at room temperature.

### Intracellular Transcription Factor Staining

FoxP3 transcription factor staining kit protocol (eBiosciences, San Diego, CA, USA) was used for intracellular transcription factor staining. In brief, T cells were stained for desired surface markers after dead cell discrimination and blocking with CD16/32 (2.4 G) antibody. After 20 min of incubation, the cells were washed twice and then fixed and permeabilized at room temperature for 20 min as recommended by the manufacturer. The cells were then washed twice with permeabilization buffer and then stained with transcription factor antibody cocktail for 20–30 min at room temperature.

### Statistical Analysis

Non-parametric Mann–Whitney test was used for comparisons between two groups. One-way ANOVA with Bonferroni’s Multiple Comparison Test was used for multiple comparisons having three or more groups. Two-way ANOVA with Bonferroni posttest was used for comparing weight lost. A log-rank (Mantel–Cox) test was used to determine the significance of survival of PbA-infected mice with or without anti-ratIgG2b and anti-ICOS treatment. Graphs depict mean values ± SEM. *p*-value < 0.05 (*p < 0.05; **p < 0.01; ***p < 0.001) was considered significant. All graphs were prepared with Prism 5.0 (GraphPad, La Jolla, CA, USA) software.

## Results

### Anti-ICOS Administration Slows *P. berghei* ANKA Growth and Prolonged Mice Survival

**Figure 1:**
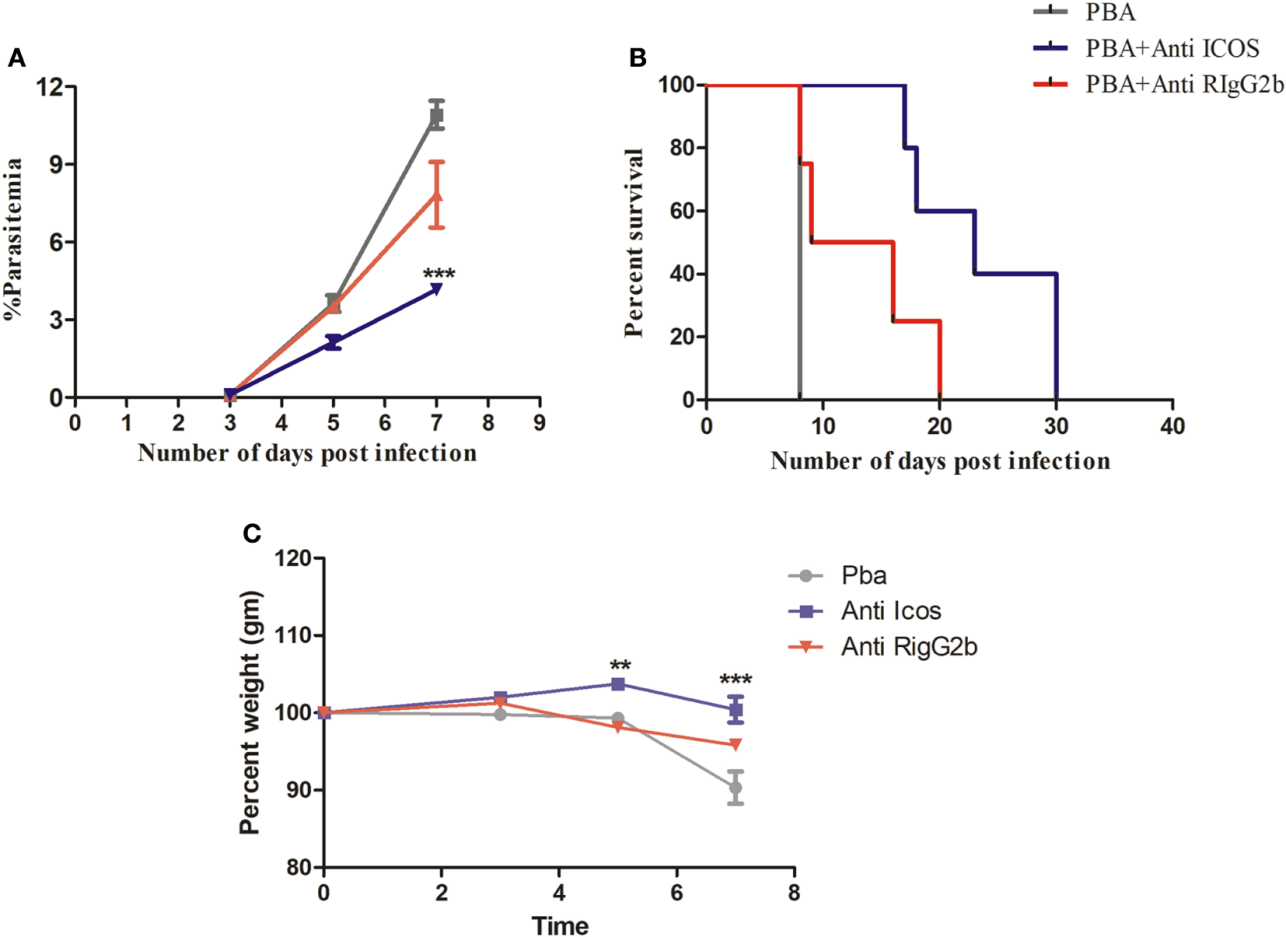
Inducible costimulator (ICOS) positively regulates *Plasmodium berghei* ANKA parasite growth and pathogenesis. BALB/c mice (*n* = 6/group) were infected with i.v. injection of 1 × 10^4^ *P. berghei* ANKA and on day 1 of infection treated with a single dose of anti-ICOS (200 μg) or isotype control (200 μg). **(A)** Percent parasitemia calculated from Geimsa-stained tail vein blood. **(B)** Percent survival of mice from *Plasmodium berghei* ANKA infection with anti-ICOS treatment and isotype control. **(C)** Percent weight loss a marker of immunopathology. Data represent one of the two independent experiments. Bar represent mean ± SEM. Statistics: Mann–Whitney test, two-way ANOVA with Bonferroni posttest was used for comparing weight loss. Log-rank (Mantel-Cox) test applied for significance of percent survival, *p*-value <0.05 (*p < 0.05; **p < 0.01; ***p < 0.001) considered significant.

To understand the possible involvement of ICOS in the lethality of blood stage *PbA* infection, we first established parasite infection in BALB/c mice by intravenous injection of 1 × 10^4^ parasitic RBCs. To one group of PbA-infected mice, a single dose of anti-ICOS was given while to another group, rat-anti-IgG2b antibody was injected. On every alternate day starting from day 3, percent parasitemia was ascertained from Giemsa-stained thin blood smear prepared from tail vein blood. On Day 3, the mean parasitemia in untreated (0.0875 ± 0.025%), anti-ICOS (0.1%), and anti-rat IgG2b (0.1%) treated mice were found to be similar, indicating that infection in all the groups started equally. On Day 5 of infection, we observed lowered percent parasitemia albeit non-significant in anti-ICOS treated mice (2.125 ± 0.478%) when compared to untreated (3.62 ± 0.643%) and rat IgG2b-treated (3.5 ± 0.408%) mice. Statistically significant lower parasitemia in anti-ICOS treated (4.16 ± 0.372, *p-*value < 0.001) as compared to untreated PbA group (10.916 ± 1.067) (Figure 1A) was established on day 7. Lowered parasitemia in anti-ICOS-treated mice suggested that ICOS indeed played a significant role in parasite growth. Further, analyzing for the survival of the mice, anti-ICOS treated mice survived significantly longer than the rat IgG2b treated and rat IgG2b treated mice survived longer than the non-treated one (Figure 1B). Eventually, anti-ICOS treated mice died with hyperparasitemia. Next, we analyzed percent weight loss; a pathological marker in anti-ICOS treated and untreated mice during PbA infection. On day 5 and day 7 of PbA infection, significantly reduced percent weight loss was observed in anti-ICOS treated mice as compared to PbA-infected mice (Figure 1C). The above results indicated that ICOS plays a significant role in PbA parasite growth and pathogenesis.

**Figure 2:**
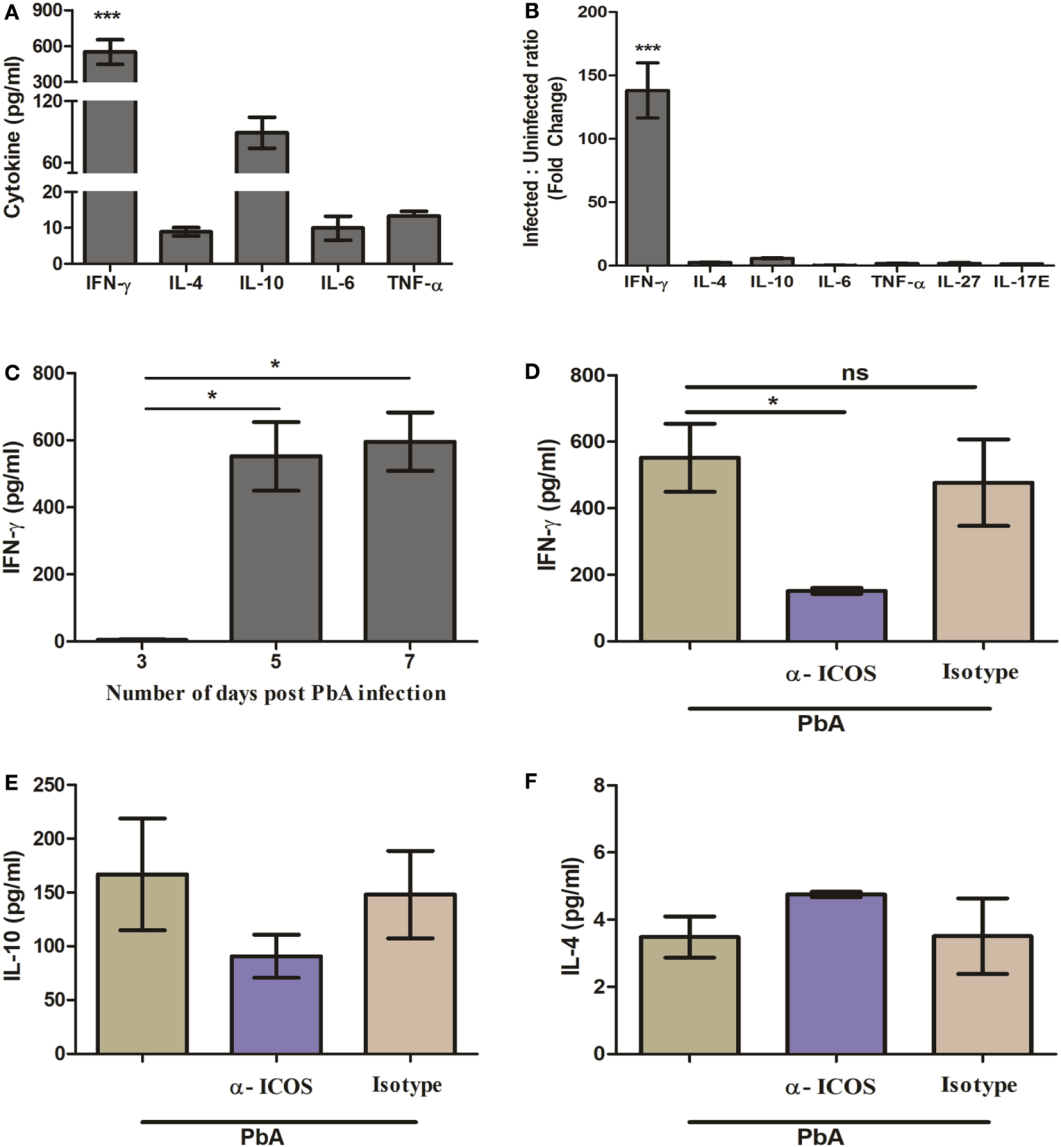
Th1 cytokine IFN-γ is highly produced during PbA infection. Plasma was collected on indicated days from anti-inducible costimulator (ICOS), anti-ratIgG2b, untreated PbA infected and uninfected (control) mice (*N* = 3/group). The collected plasma was analyzed for cytokine IFN-γ, IL-4, IL-10, IL-6, TNF-α, IL-27, and IL-17E with bioplex ELISA. **(A)** Th1 cytokine IFN-γ is significantly increased as compared to Th2 (IL-4), Treg (IL-10), TNF-α, and IL-6 in *Plasmodium berghei* ANKA (PbA)-infected mice. **(B)** Percent cytokine change as compared to uninfected mice. **(C)** Kinetics of IFN-γ during PbA infection. **(D)** Reduction in plasma IFN-γ production by anti-ICOS treatment. **(E)** Plasma level of IL-10 **(F)** plasma level of IL-4. Data represent one of the two independent experiments. Bar represent mean ± SEM. Statistics: Mann–Whitney test, one-way ANOVA with Bonferroni’s Multiple Comparison Test, *p-*value <0.05 (*p < 0.05; **p < 0.01; ***p < 0.001) considered significant.

It is well known that regulation of pro-inflammatory (IFN-γ, TNF-α, IL-6, IL-17) and anti-inflammatory (IL-10, IL-4) cytokines play a pivotal role in malaria parasite growth, protection, and/or pathogenesis (28–32). Moreover, previous studies in blood stage infection of PbA have determined the crucial role of IFN-γ in immunopathology and death. However, during acute non-lethal blood-stage malaria infection, IL-27-mediated IL-10 production from IFN-γ-producing CD4^+^ T cells has been shown to play an essential role in protection from immune pathology (33). Thus, we scored for plasma cytokine IFN-γ, IL-4, IL-10, IL-17, IL-6, IL-27, and TNF-α during PbA infection in BALB/c mice with and without anti-ICOS treatment. In plasma samples of PbA-infected mice, significantly higher IFN-γ levels were observed as compared to other tested cytokines (Figure 2A). The ratio of cytokines in infected to uninfected samples (fold change) also suggested that IFN-γ production was higher than other tested cytokines between the groups (Figure 2B). Moreover, significantly higher IFN-γ was observed on day 5 and 7 of PbA infection as compared to day 3 (Figure 2C). Interestingly, significantly reduced plasma IFN-γ level was found after anti-ICOS treatment (Figure 2D). Also, non-significant changes in the levels of cytokine IL-10 (Figure 2E), IL-4 (Figure 2F), and no difference in the levels of other tested cytokines were observed (data not shown). Altogether, this suggested the critical involvement of ICOS in malaria parasite growth and lethality might be through IFN-γ production.

### ICOS Expression During Blood Stage *P. berghei* ANKA Infection

**Figure 3:**
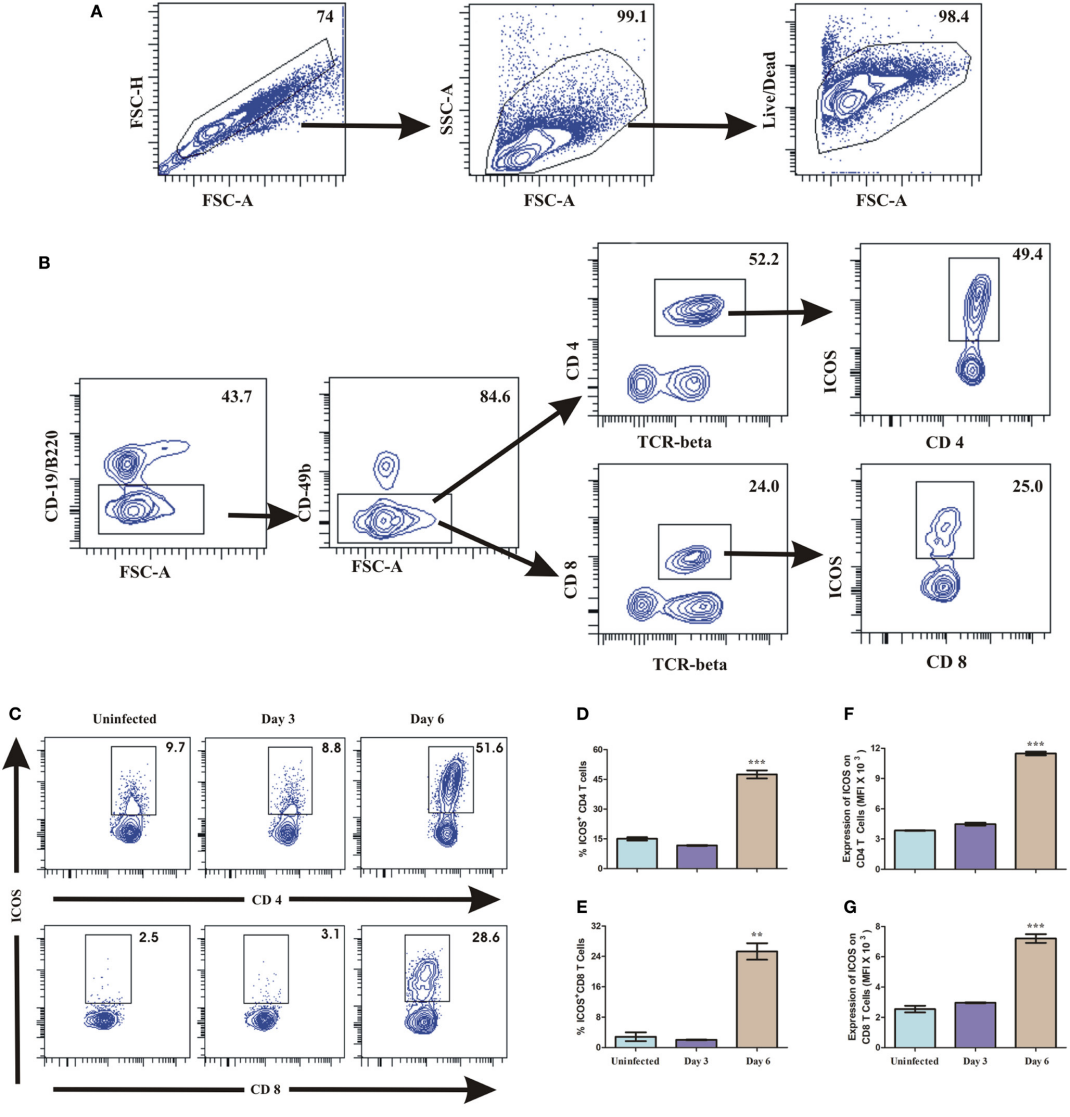

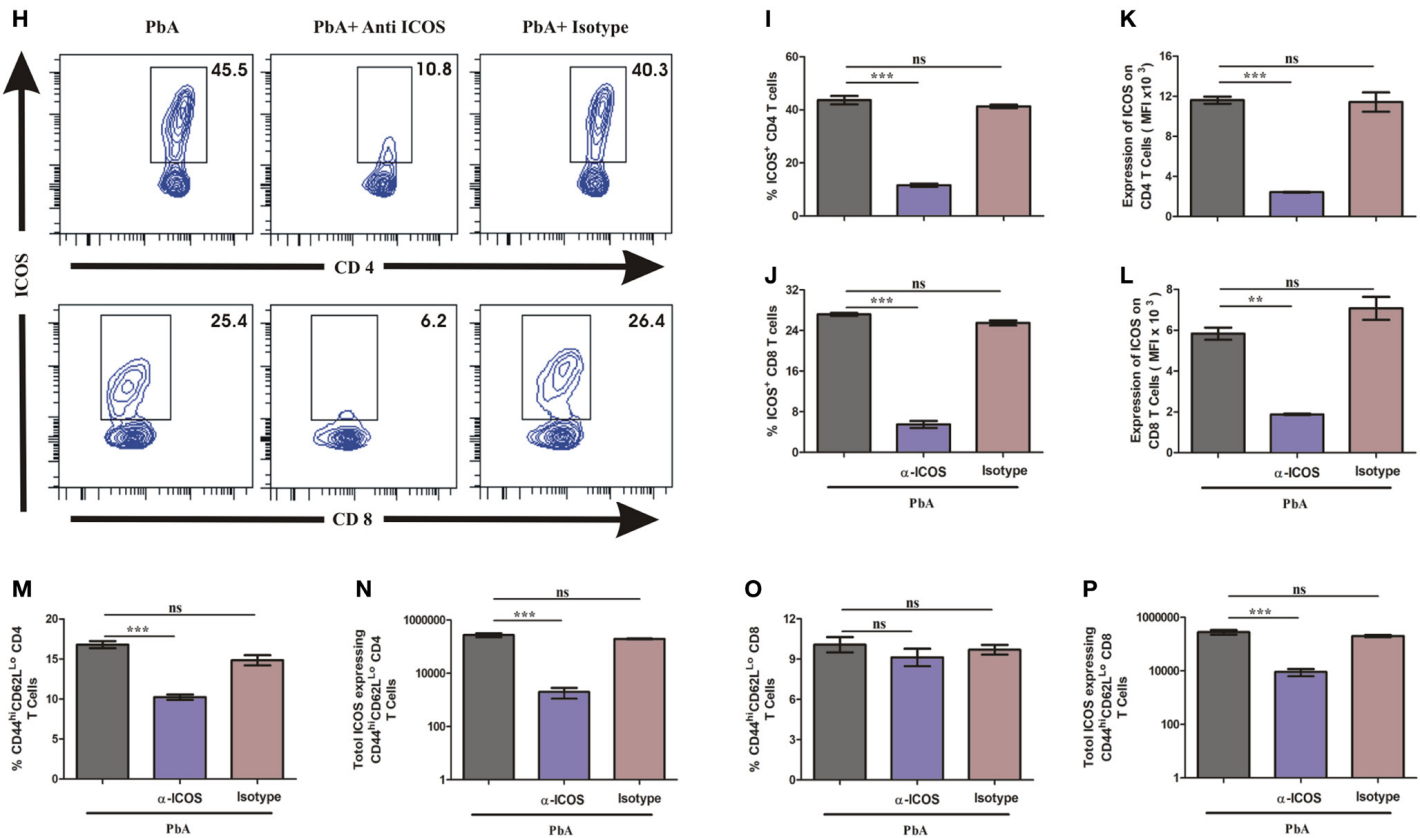
Both CD4^+^ and CD8^+^ T cells express inducible costimulator (ICOS) during *Plasmodium berghei* ANKA (PbA). A single cell suspension of splenic cells from uninfected and PbA infected (*N* = 3/group) were stained with surface staining with antibodies to anti-TCR-vb, anti-CD4, anti-CD8a, CD19/B220, CD278 (ICOS) after dead cell staining with fixable blue dead cell stain. **(A)** Gating strategy for selection of live cells after doublet and dead cell discrimination. This gating strategy was used for all flow cytometry analysis. **(B)** Representative gating strategy for selecting CD4^+^ and CD8^+^ T cells and ICOS expression. **(C)** Representative counter plot showed ICOS expression by CD4^+^ and CD8^+^ T cells on day 3 and day 6 of PbA infection. **(D)** Percentage ICOS expressing CD4^+^ T cells. **(E)** Percentage ICOS expressing CD8^+^ T cells. **(F)** Expression of ICOS on CD4^+^ T cells. **(G)** Expression of ICOS on CD8^+^ T cells. **(H)** Representative counter plot showed depletion of ICOS expressing CD4^+^ and CD8^+^ T cells upon anti-ICOS treatment. **(I)** Percentage depletion of ICOS expressing CD4^+^ T cells. **(J)** Percentage depletion of ICOS expressing CD8^+^ T cells. **(K)** ICOS expression on non-depleted CD4^+^ T cells. **(L)** ICOS expression on non-depleted CD8^+^ T cells. **(M)** Percentage activated (CD44^hi^CD62L^Lo^) CD4^+^ T cells. **(N)** Total number of ICOS expressing activated (CD44^hi^CD62L^Lo^) CD4^+^ T cells. **(O)** Percentage activated (CD44^hi^CD62L^Lo^) CD8^+^ T cells. **(P)** Total number of ICOS expressing activated (CD44^hi^CD62L^Lo^) CD8^+^ T cells. Data represent one of the three independent experiments. Bar represent mean ± SEM. Statistics: Mann–Whitney test, one-way ANOVA with Bonferroni’s Multiple Comparison Test, *p*-value <0.05 (*p < 0.05; **p < 0.01; ***p < 0.001) considered significant.

To investigate the role of ICOS and cells associated with its expression for *PbA* lethality, we scored ICOS expression on lymphocytes. On day 3 and day 6 of *PbA* infection, mice were sacrificed and their spleens analyzed for surface markers to identify ICOS expression on CD4^+^, CD8^+^, NK, NKT, and B cells by flow cytometry. After gating out doublets and dead cells (Figure 3A), we observed higher ICOS expression on CD4^+^ and CD8^+^ T cells (Figure 3B). Minimal ICOS expression was found on B cells, NK cells, and NKT cells in PbA infected and uninfected mice (data not shown) that did not vary with progressive infection. On day 6 of infection, however, we observed an increased percentage of ICOS-positive CD4^+^ and CD8^+^ T cells as compared to uninfected mice (Figures 3C–E). A significant increase in mean fluorescent intensity for ICOS on CD4^+^ and CD8^+^ T cells suggested that expression of ICOS at protein level per cell was also higher on day 6 of PbA infection (Figures 3F,G). Anti-ICOS treatment significantly reduced the percentage of ICOS-positive CD4^+^ and CD8^+^ T cells (Figures 3H–J) while ICOS MFI on non-depleted T cells from anti-ICOS treated mice was significantly lower (Figures 3K,L) than untreated and RatIgG-treated mice. Moreover, we observed significant depletion in percent activated (CD44^hi^CD62L^lo^) as well as total ICOS expressing CD44^hi^CD62L^lo^ CD4^+^ T cells upon anti-ICOS treatment (Figures 3M,N). Further, we observed non-significant depletion in percent activated (CD44^hi^CD62L^lo^) CD8^+^ T cells and a significant depletion in ICOS expressing CD44^hi^CD62L^lo^ CD8^+^ T cells (Figures 3O,P). However, anti-Rat IgG2b administration non-significantly reduced ICOS expressing CD4^+^ and CD8^+^ T cells (Figures 3I,K,N,P). Collectively, the above data indicated that PbA growth and lethality might be associated with ICOS expressing CD4^+^ and CD8^+^ T cells.

### IFN-γ-Producing CD4^+^ T Cells Are Highly ICOS Positive at Protein Level

**Figure 4:**
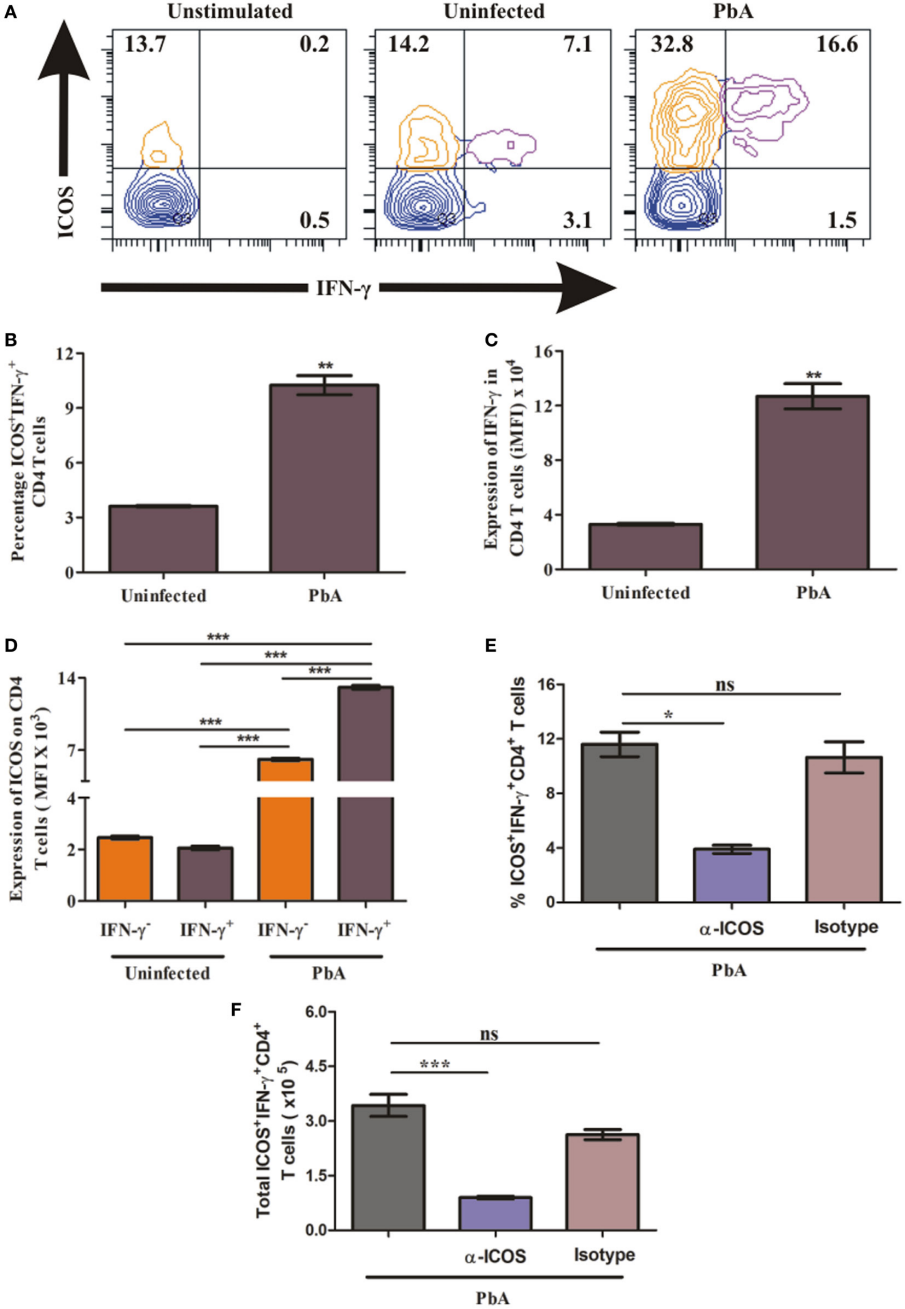

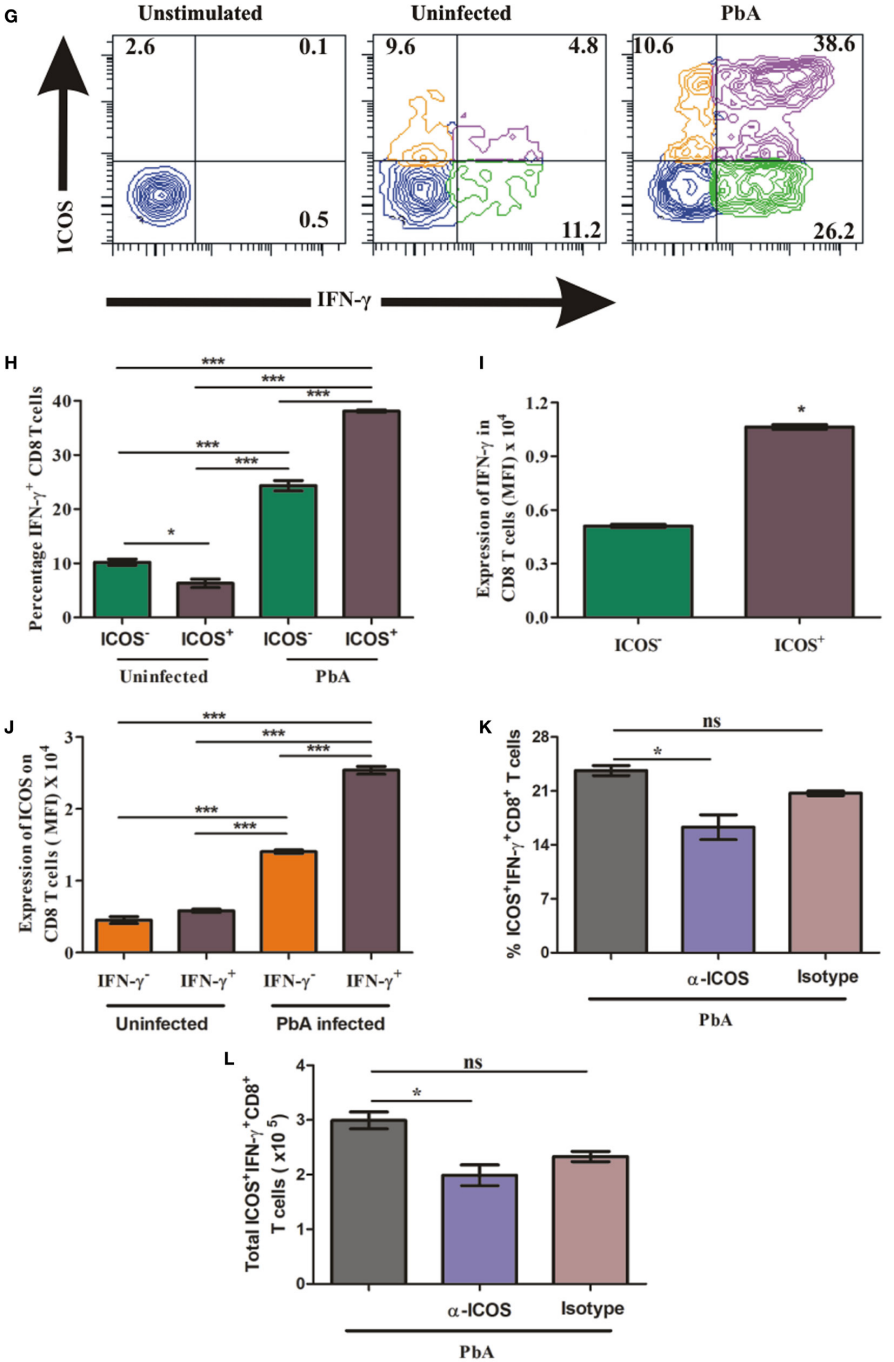
Higher inducible costimulator (ICOS) expression on IFN-γ producing T cells. Negatively isolated CD4^+^ T cells and single cell splenocyte from *Plasmodium berghei* ANKA infected and uninfected mice (*N* = 5/group) were stimulated by Phorbol 12-myristate 13-acetate/Ionomycin for 5 h with Brefeldin A at last 2.5 h. Stimulated CD4^+^ T cells were stained with anti-ICOS and anti-IFN-γ after dead cell discriminator staining and Fc blocking. The stimulated single cell splenocytes were stained with anti-TCR-vb, anti-CD8a, anti-ICOS, and IFN-γ. **(A)** Representative counter plot show co-expression of ICOS and IFN-γ by CD4^+^ T cells. **(B)** Percentage increase in ICOS^+^IFN-γ^+^ CD4^+^ T cells. **(C)** Increased expression of IFN-γ in CD4^+^ T cells. **(D)** ICOS expression on IFN-γ^+^ and IFN-γ^−^ CD4^+^ T cells. **(E)** Anti-ICOS treatment reduced percentage ICOS^+^IFN-γ^+^ CD4^+^ T cells. **(F)** Anti-ICOS treatment depleted total number of ICOS^+^IFN-γ^+^ CD4^+^ T cells after anti-ICOS treatment. **(G)** Representative counter plot show expression of ICOS and IFN-γ in CD8^+^ T cells. **(H)** Percentage IFN-γ positive CD8^+^ T cells in ICOS^+^ and ICOS^−^ population. **(I)** Expression of IFN-γ by ICOS^+^ and ICOS^−^ CD8^+^ T cells. **(J)** ICOS expression on IFN-γ^+^ and IFN-γ^−^ CD8^+^ T cells. **(K)** Anti-ICOS treatment reduced percentage ICOS^+^IFN-γ^+^ CD8^+^ T cells. **(L)** Anti-ICOS treatment depleted total number of ICOS^+^IFN-γ^+^ CD8^+^ T cells. Data represent one of the two independent experiments. Bar represent mean ± SEM. Statistics: Mann–Whitney test, one-way ANOVA with Bonferroni’s multiple comparison test, *p*-value <0.05 (*p < 0.05; **p < 0.01; ***p < 0.001) considered significant.

Higher ICOS expression on T cells and increased plasma IFN-γ production suggested that ICOS expressing T cells may be the source and major producers of IFN-γ. Thus, to quantify IFN-γ production by ICOS expressing CD4^+^ T cells, on day 6, we negatively isolated CD4^+^ T cells from uninfected and *PbA* infected BALB/c mice and stimulated them with PMA/ionomycin. The stimulated cells were analyzed for ICOS and IFN-γ co-expression by staining cells with anti-ICOS, anti-IFN-γ antibodies after dead cell discrimination and Fc blocking (Figure 4A). As expected, we found a significant increase in the percentage of ICOS^+^IFN-γ^+^ CD4^+^ T cells from *PbA-*infected mice as compared to uninfected mice (Figure 4B). Increased integrated mean fluorescent intensity for IFN-γ depicted that IFN-γ production was significantly higher in CD4^+^ T cell from infected mice than in uninfected mice at the protein level (Figure 4C). We further analyzed ICOS expression on IFN-γ positive and IFN-γ negative CD4^+^ T cells from PbA-infected mice. We found that ICOS expression per cell (as scored with mean fluorescent intensity) was significantly higher on IFN-γ^+^ cells than the negative ones (Figure 4D). Moreover, anti-ICOS treatment significantly reduced percentage as well as total ICOS^+^IFN-γ^+^ CD4^+^ T cells (Figures 4E,F). Taken together, our data for CD4^+^ T cells suggest that ICOS may play a role in IFN-γ production based on correlation of ICOS expression and IFN-γ production. It also clearly indicated that all IFN-γ-producing CD4^+^ T cells were ICOS positive but all ICOS-positive CD4^+^ T cells were not IFN-γ producers.

### ICOS Expressing CD8^+^ T Cells Are Higher IFN-γ Producers

To ascertain the role of ICOS in the production of IFN-γ by CD8^+^ T cells during PbA infection, we stimulated single cell splenocyte with PMA/ionomycin. The stimulated cells were stained with anti-TCR-Vb, anti-CD8a, anti-ICOS, and anti-IFN-γ antibodies. After gating TCR-Vb and CD8^+^, we observed four populations including ICOS^+^IFN-γ^−^, ICOS^+^IFN-γ^+^, ICOS^−^IFN-γ^+^, and ICOS^−^IFN-γ^−^ CD8^+^ T cells (Figure 4G). We observed that all ICOS-expressing CD8^+^ T cells were not IFN-γ producers and all IFN-γ producers were not ICOS expressing (Figure 4G). Additionally, we found a significant increase in percent ICOS^+^IFN-γ^+^ CD8^+^ T cells in PbA-infected mice as compared to both the CD8^+^ T cell populations (ICOS^−^IFN-γ^+^, ICOS^+^IFN-γ^+^) of uninfected mice (4H). Moreover, in PbA-infected mice, the percentage of ICOS^+^IFN-γ^+^ CD8^+^ T cells was significantly higher than the ICOS^−^IFN-γ^+^ population (Figure 4H). Increased mean fluorescence intensity of IFN-γ in ICOS^+^ CD8^+^ T cells than ICOS^−^ depicted that ICOS expressing CD8^+^ T cells were higher IFN-γ producers (Figure 4I). Moreover, ICOS expression in PbA-infected mice was higher on ICOS^+^IFN-γ^+^ CD8^+^ T cells than ICOS^+^IFN-γ^−^ CD8^+^ T cells as determined by the increased mean fluorescent intensity (Figure 4J). In addition, we found anti-ICOS treatment significantly reduced percentage as well as total ICOS^+^IFN-γ^+^ CD8^+^ T cells (Figures 4K,L). Taken together, the above results suggested that during PbA infection, IFN-γ was produced by ICOS^+^ and ICOS^−^ CD8^+^ T cells; however, ICOS^+^ CD8^+^ T cells were higher producers of IFN-γ than the ICOS^−^ ones.

### T-Bet Expressing CD4^+^ and CD8^+^ T Cells Have Higher ICOS

**Figure 5:**
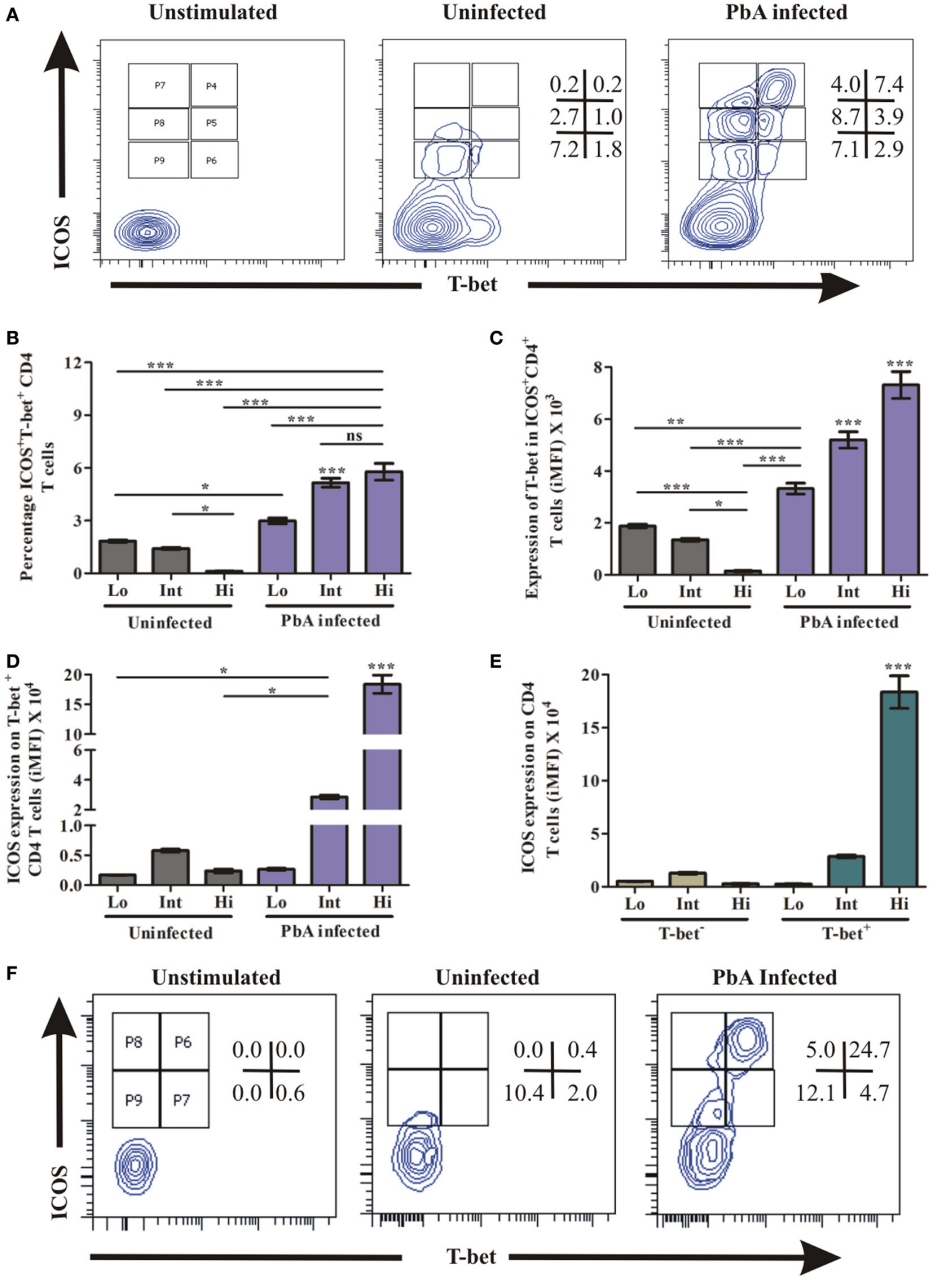

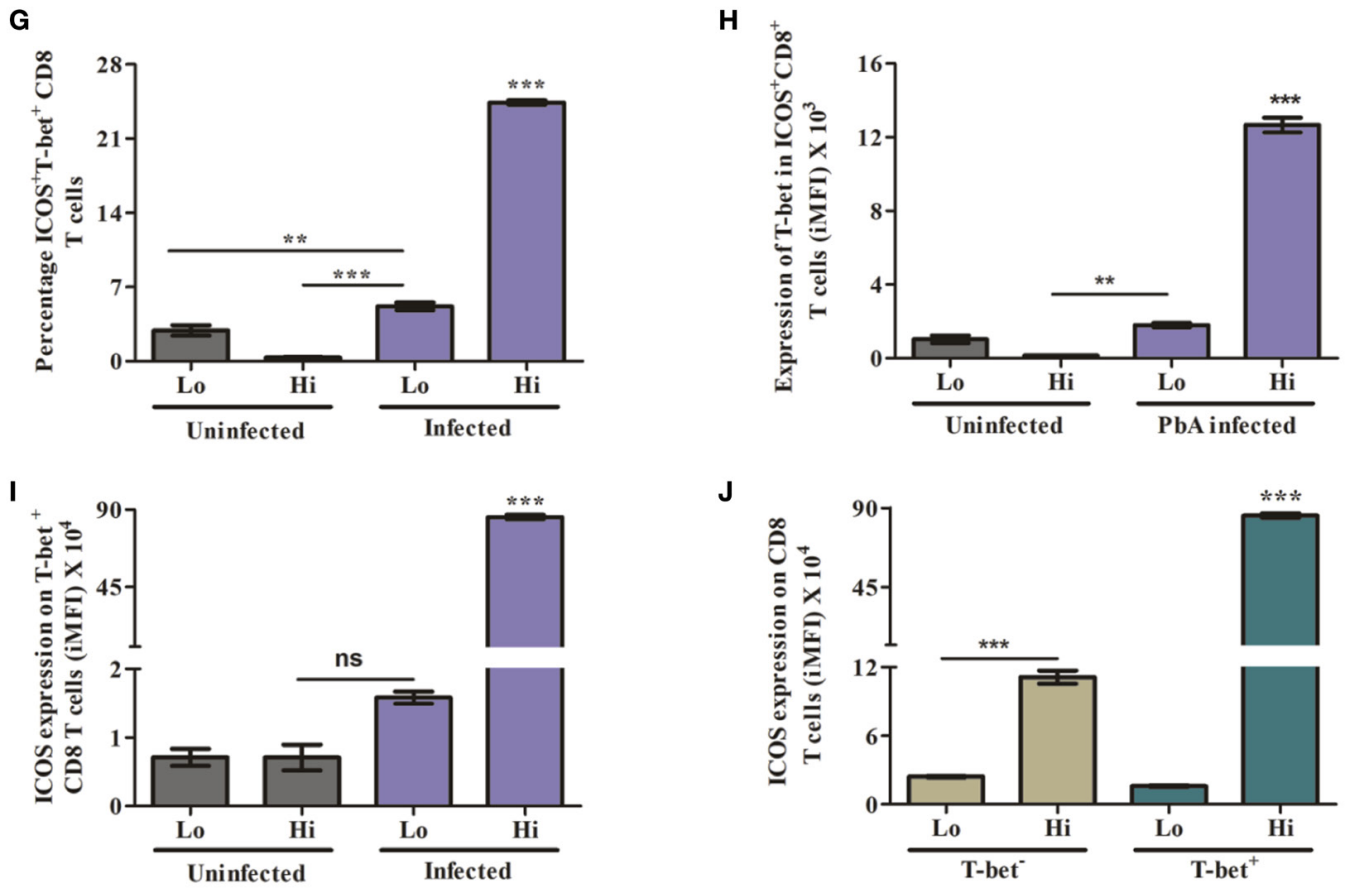
Higher inducible costimulator (ICOS) expression on T-bet-positive T cells. Negatively isolated CD4^+^ T cells and single cell splenocyte from *Plasmodium berghei* ANKA infected and uninfected mice (*N* = 3/group) were stimulated by Phorbol 12-myristate 13-acetate/ionomycin for 5 h. Stimulated CD4^+^ T cells were stained with anti-ICOS and anti-T-bet antibodies after dead cell staining and Fc blocking. The stimulated single cell splenocytes were stained with anti-TCR-vb, anti-CD8a, anti-ICOS, and T-bet. **(A)** Representative counter plot show co-expression of ICOS and T-bet by CD4^+^ T cells. **(B)** Percentage increase in ICOS^+^T-bet^+^ CD4^+^ T cells. **(C)** Increased expression of T-bet in ICOS^+^CD4^+^ T cells. **(D)** Increased ICOS expression on T-bet positive CD4^+^ T cells. **(E)** ICOS expression on T-bet^+^ and T-bet^−^ CD4^+^ T cells. **(F)** Representative counter plot show expression of ICOS and T-bet in CD8^+^ T cells. **(G)** Percentage T-bet positive CD8^+^ T cells in ICOS^+^ and ICOS^-^ population. **(H)** Expression of T-bet by ICOS^+^CD8^+^ T cells. **(I)** ICOS Expression on T-bet expressing CD8^+^ T cells. **(J)** ICOS expression on T-bet^+^ and T-bet^−^ CD8^+^ T cells. Data represent one of the two independent experiments. Bar represent mean ± SEM. Statistics: Mann–Whitney test, one-way ANOVA with Bonferroni’s multiple comparison test, *p-*value < 0.05 (*p < 0.05; **p < 0.01; ***p < 0.001) considered significant.

The master transcription factor T-bet importantly involved in regulating IFN-γ has been shown to play a paradoxical role during blood-stage malaria infection. As an example, in non-lethal blood stage infection, transcription factor T-bet plays a role in parasite growth wherein T-bet knock out mice infected with *Plasmodium yoelii yoelii* exhibited lower parasitemia (34). Conversely, during PbA infection, while T-bet regulated parasite burden, it also promoted ECM pathology (11). Moreover, T-bet expression critically depends on ICOS expression and its downstream signaling (35). Therefore, we hypothesized that ICOS expressing CD4^+^ and CD8^+^ T cells promote PbA parasite growth through T-bet expression. Thus, we analyzed co-expression of ICOS and T-bet by CD4^+^ and CD8^+^ T cells during blood stage PbA infection. To ascertain ICOS and T-bet co-expression, we negatively isolated CD4^+^ T cells and stimulated with PMA/ionomycin for 5 h. The stimulated CD4^+^ T cells were stained with anti-ICOS and anti-T-bet antibody. We observed low (Lo), intermediate (Int), and high (Hi) ICOS^+^T-bet^+^ and ICOS^+^T-bet^−^ CD4^+^ T cell population (Figure 5A) in *PbA*-infected mice. Percent ICOS^+^T-bet^+^ (Lo), ICOS^+^T-bet^+^ (Int) CD4^+^ T cells were significantly higher than ICOS^+^T-bet^+^(Hi) in uninfected mice. Whereas in PbA infected mice, percent ICOS^+^T-bet^+^(Hi) and ICOS^+^T-bet^+^(Int) were significantly higher than ICOS^+^T-bet^+^(Lo) of *PbA*. Percent ICOS^+^T-bet^+ (Hi,Int,Lo)^ CD4^+^ T cells of infected mice was higher than ICOS^+^T-bet^+ (Hi,Int,Lo)^ of the uninfected (Figure 5B). We observed significantly higher T-bet expression in CD4^+^ T cells from PbA-infected mice as compared to the uninfected (Figure 5C). Also, we analyzed ICOS expression on T-bet-positive CD4^+^ T cells from infected and uninfected mice. We observed significantly higher ICOS expression on T-bet^(Int, Hi)^ positive CD4^+^ T cells of PbA-infected mice as compared to T-bet^+ (Hi,Int,Lo)^ of uninfected and T-bet (low) of infected mice (Figure 5D). ICOS expression was significantly higher in T-bet^+^ (Hi) than T-bet^−^ CD4^+^ T cells of PbA-infected mice (Figure 5E). Thus, our data suggested that all T-bet expressing CD4^+^ T cells were ICOS positive, but all ICOS-positive CD4^+^ T cells were not expressing T-bet.

Similar to CD4^+^ T cells, we determined the role of ICOS for T-bet expression in CD8^+^ T cells during PbA infection. The splenocytes were stimulated as described earlier and stained with TCR-Vb, CD8a, ICOS, and T-bet antibodies. After gating TCR-vb and CD8^+^, we observed four populations; ICOS^+^T-bet^+ (Hi)^, ICOS^+^T-bet^+ (Lo)^, ICOS^+^T-bet^− (Hi)^, and ICOS^+^T-bet^− (Lo)^ of CD8^+^ T cells (Figure 5F). A significant increase in percent ICOS^+^T-bet^+(Hi)^ CD8^+^ T cells was observed as compared to ICOS^+^T-bet^+ (Lo)^ of infected mice. Also, it was higher than the uninfected (ICOS^+^T-bet^+ (Hi, Lo)^) CD8^+^ T cell population (Figure 5G). Expression of T-bet at protein level per cell was significantly higher in ICOS^+^T-bet^+ (Hi)^ than ICOS^+^T-bet^+ (Lo)^ in PbA infected mice and ICOS^+^T-bet^+ (Hi)^, ICOS^+^T-bet^+ (Lo)^ CD8^+^ T cells in uninfected mice (Figure 5H). Also, we analyzed ICOS expression on T-bet positive cells from uninfected and PbA infected mice and found that ICOS expression was higher on ICOS^+^T-bet^+^^(Hi)^ (Figure 5I). Moreover, ICOS expression on T-bet positive CD8^+^ T cells was higher than T-bet negative cells in infected mice (Figure 5J). Thus, our data suggested that all T-bet expressing CD8^+^ T cells were ICOS positive, but all ICOS positive CD8^+^ T cells were not expressing T-bet. Taken together, our data indicated that the transcription factor T-bet was expressed by ICOS positive CD4^+^ as well as CD8^+^ T cells during PbA infection.

## Discussion

Most symptoms such as fever, anemia, and cerebral manifestations occur during the asexual erythrocytic or blood-stage malaria parasite infection and have been shown to be strongly associated with IFN-γ production by various cells. The produced IFN-γ is involved in modulation of parasite growth leading to protection as well as pathology (7, 8, 29, 36–38). In experimental allergic encephalomyelitis, IFN-γ-mediated immunopathology is associated with ICOS expression and its signaling (39). Further, impaired IFN-γ production and reduced CD4^+^ and CD8^+^ T cell response was observed in ICOS-deficient patients leading to immunodeficiency and autoimmunity (40, 41). Moreover, during non-lethal blood-stage malaria infection, ICOS plays an essential role as a regulator of IFN-γ production and also in distributing parasite-specific CD4^+^ T cells to both lymphoid and non-lymphoid organs (20, 42). Thus, these studies suggested that ICOS could play a role in IFN-γ production during infectious and autoimmune diseases (43). ICOS signaling regulate transcription factor T-bet, which is also involved in regulation of malaria parasite growth and pathogenesis (11, 34, 35). Taking these studies into consideration, we tried to demonstrate the role of ICOS in parasite growth and lethality through T-bet expression and IFN-γ production, during lethal blood stage of PbA infection. Thus, in our initial experiment, the depletion of ICOS-expressing cells resulted in significantly lowered parasitemia and prolonged mice survival, suggesting that ICOS positive cells are required for PbA growth. Lowered parasitemia due to substantial slowing of parasite maturation, was observed during intraerythrocytic *P. berghei* ANKA infection in Rag^(−/−)^ (lacks T and B cells and mount weak inflammatory response) mice (44). Thus, in our study, we speculate that impaired T cell response leading to diminished parasitemia might be associated with parasite maturation. Further, reduced percent weight loss (a marker of pathology) in anti-ICOS treated mice as compared to untreated suggested that ICOS expressing cells are also involved in PbA pathology. The role of ICOS in parasite growth and pathology, thus, led us to further characterize its expression during PbA infection.

During blood-stage malarial infection, helper CD4^+^ and cytotoxic CD8^+^ T cells contribute to both protection and pathology (45–48). For activation of both T cells, ICOS expression, and its downstream signaling is critical (21, 40, 41, 49, 50). Consistent with this, in our experiment during blood stage PbA infection, ICOS may be involved in the activation of splenic CD4^+^ and CD8^+^ T cells as both these cells express significantly higher ICOS. A study involving depletion of these T cells with antibodies prevented pathology of experimental cerebral malaria (5). Similarly, in this study, upon anti-ICOS administration, depletion of ICOS expressing CD4^+^ and CD8^+^ T cells led to lower parasitemia, longer survival, and ameliorated pathology. Thus our study suggested that depletion of ICOS expressing CD4^+^ and CD8^+^ T cell was sufficient to control parasite growth and lethality as compared to total T cell depletion.

IFN-γ has been known to play a critical role in lethality during *P. berghei* ANKA infection (9) and consistent with these earlier findings, increased plasma IFN-γ production in our study may be correlated with its role in PbA pathogenesis. Lowered parasitemia, reduced IFN-γ, and increased survival upon anti-ICOS treatment suggest that ICOS plays a role in PbA growth and lethality through IFN-γ production. Further, characterization of CD4^+^ and CD8^+^ T cells for IFN-γ production showed that both these cells were indeed IFN-γ producers. Moreover, depletion of ICOS-expressing CD4^+^ and CD8^+^ T cells and reduction of plasma IFN-γ upon anti-ICOS treatment suggested that in blood stage of PbA infection, ICOS expressing CD4^+^ and CD8^+^ T cells were the major producers of IFN-γ. Further, earlier studies also demonstrated the role of ICOS in modulating cytokines of naïve and activated T cells. Moreover, during *Mycobacterium tuberculosis*, ICOS signaling controlled antigen-specific protective IFN-γ production and the produced IFN-γ, in turn, regulated ICOS expression (24). Similarly, in our study, higher ICOS expression on IFN-γ producing T cells and higher IFN-γ production in ICOS expressing T cells suggested that both ICOS and IFN-γ could regulate each other.

On similar line master transcription factor T-bet has shown to be involved in malaria parasite growth and pathogenesis. For example, lower parasite burden was observed in T-bet knock out (Tbx21^−/−^) mice than T-bet wild-type mice suggesting that T-bet promotes malaria parasite growth (34). In another study, T-bet regulated PbA growth but has also been shown to promote the pathogenesis of ECM (11). Interestingly, ICOS expression and its downstream signaling have been shown to be critical for T-bet expression (35). In our study, ICOS expressing CD4^+^ and CD8^+^ T cells, which were involved in PbA growth has also been shown to co-express T-bet. Our results also demonstrated that ICOS and T-bet might regulate each other’s expression as correlated with higher ICOS expression on T-bet positive cells and *vice versa*.

The malaria parasite has evolved with multiple mechanisms such as changing shapes, motility, metabolic requirement, and immune evasion strategies to survive inside the host (51). In correlation with this, we demonstrated that malaria parasite utilizes ICOS-expressing T cells for their growth through T-bet expression and IFN-γ production. Other studies have depicted that altering ICOS signaling leads to modulated T cell response, which in turn is involved in infection clearance, tumor regression, and ameliorated pathology (52–54). Thus, modulation of ICOS expression and its signaling might be helpful in altering parasite growth and lethality, which might be valuable in preventing severe malaria in humans.

## Ethics Statement

The use of animals and animal procedures were approved by the Institutional Animal Ethics Committee, Institute of Life Sciences, Bhubaneswar, India in accordance with the “Committee for the Purpose of Control and Supervision of Experiments on Animals (CPCSEA).”

## Author Contributions

GJ conceived the study and discussed with SD. GJ planned and performed experiments, analyzed data, and drafted manuscript. SS, GB, SS, and PB helped to perform components of some of the experiments. SD arranged the grants for the study and edited manuscript. All authors contributed intellectual content and approved it for publication.

## Conflict of Interest Statement

The authors declare that the research conducted have no commercial or financial involvement that could be considered as potential conflict of interest.
